# Non-ablative dermis layer targeted dielectric heating for facial rhytid improvement in Asian skin

**DOI:** 10.1016/j.jpra.2026.02.014

**Published:** 2026-02-18

**Authors:** Kyuho Yi, Ohdeog Kwon, Yerin Park, Ohara Natsue, Kaveh Karandish, Eric Sooyoung Ahn, Ti Jo Tsay

**Affiliations:** a You & I Clinic, Seoul, Korea; b Half Clinic, Seoul, Korea; c Medical Research, Seoul, Korea; d Natsu Clinic, Hyōgo, Japan; e PCH Medspa, California, USA; f Eric S. Ahn Medical Corporation, California, USA; g Ageless MD, California, USA

**Keywords:** Dielectric-field heating, Low-temperature thermal shock, Nasolabial fold, Marionette line, Facial rejuvenation

## Abstract

**Background:**

Low-temperature dielectric-field heating has emerged as a non-ablative modality that selectively engages water-rich dermal and fibro-septal structures while sparing adipose tissue. Operating within a controlled thermal-shock window (∼42–45 °C), the Dermis Layer Targeted Dielectric Heating System (DLTD) —when utilized at low-to-moderate intensity levels—induces reversible collagen recoil and early dermal tightening without high-temperature injury. Although conceptually suited for mid- and lower-face rhytids, clinical data remain limited.

**Objective:**

To evaluate the clinical efficacy, three-dimensional structural elevation, and safety of a single DLTD session, applied within a low-to-moderate energy range, for improving nasolabial folds and marionette lines in Asian women.

**Methods:**

Thirty-two women aged 35–65 years with visible nasolabial folds and marionette lines received one DLTD session delivered at low-to-moderate intensity levels along the midface-to-lower-face axis. Outcomes were evaluated at baseline, immediately after treatment, and at weeks 4 and 8. Wrinkle severity was assessed using WSRS and the Merz scale, and structural changes were quantified by 3D vector analysis of midface elevation and marionette descent. GAIS ratings were obtained at week 8, and pain and adverse events were documented.

**Results:**

WSRS improved from 3.1 to 2.0 (35.5%), and Merz scores from 2.6 to 1.9 (26.9%) at week 8. Three-dimensional analysis showed progressive vertical elevation and improved perioral support, with midface elevation increasing from +0.6 mm to +1.9 mm and marionette descent improving from −0.4 mm to −1.2 mm. At week 8, 95% of investigator and 92% of patient GAIS ratings indicated improvement. Pain was minimal (0.4/10), and no serious adverse events occurred.

**Conclusion:**

A single DLTD session at low-to-moderate intensity produced consistent wrinkle reduction, measurable three-dimensional structural elevation, and high satisfaction with negligible discomfort and no downtime. These findings support DLTD as a promising non-ablative option for mid- and lower-face rejuvenation. Larger controlled studies are needed to establish long-term durability and comparative efficacy.

## Introduction

Facial aging in the mid- and lower-face is driven by progressive alterations in collagen hydration, weakening of the retinacula cutis (RC), elongation of the fibro-septal network (FSN), and the gravitational displacement of malar and perioral fat. Chronologically controlled studies of dermal water content demonstrate that aging skin shows a marked decline in bound-water fraction and hydration-dependent viscoelasticity, reflecting disruptions in the collagen–water matrix that reduce its thermomechanical responsiveness.^1^ These hydration-related changes weaken collagen recoil under controlled heating and contribute to deepening of the nasolabial folds and marionette lines.

**Figure 1:**
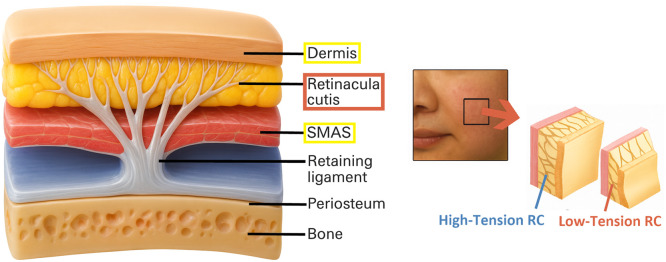
Layered skin structure and tension-bearing role of the retinacula cutis (RC). The illustration shows the dermis, retinacula cutis (RC), and SMAS, highlighting RC as vertical fibroseptal supports that anchor the dermis to deeper layers. Reduced RC density or elongation weakens vertical support, contributing to soft-tissue descent and wrinkle formation.

Histologic and imaging studies of the RC network show thinning, elongation, and regional tension loss in the anchoring pillars along the midface–perioral axis, resulting in inferior migration of the malar fat pad and increased prominence of the nasolabial fold and perioral descent.^2^ Figure 1 illustrates the layered dermis–RC–SMAS anatomy and depicts high-tension versus low-tension RC configurations, visually showing the reduction in vertical support associated with midface descent. The combined failure of collagen hydration and RC architecture forms the structural basis of mid- and lower-face aging.

Thermal-based rejuvenation technologies have attempted to counteract these aging vectors by inducing collagen remodeling. High-intensity focused ultrasound (HIFU) produces sharply confined zones of thermal coagulation but routinely reaches temperatures of 60–70 °C, which have been associated with pain, persistent erythema, susceptibility of subcutaneous fat to thermal damage, and potential contour loss.^3^ Monopolar and unipolar radiofrequency (RF) systems, by contrast, rely on resistive heating, often demonstrating slow temperature ramp-up and difficulty maintaining stable therapeutic thresholds over curved facial contours.

Low-temperature dielectric-field heating offers a different thermal paradigm. Rather than relying on high temperatures or resistive conduction, this approach uses a FocusPolar dielectric field configuration that preferentially heats tissues with high permittivity and water content. COMSOL-based modeling has demonstrated that this configuration generates convergent, volumetric electromagnetic fields capable of selectively heating the mid-dermis, FSN, and superficial musculoaponeurotic system (SMAS) while maintaining substantially lower temperatures in the epidermis and water-poor adipose tissue.^4^ Broader thermal literature on dielectric and ultra high frequency-based heating confirms that tissues with higher water fractions absorb electromagnetic energy more efficiently, producing selective volumetric heating distinct from the patterns observed with RF or HIFU.^5^

Thermal-shock research further suggests that brief exposure to temperatures around ∼45 °C for approximately 8–10 s induces collagen triple-helix recoil, early collagen-fiber tightening, and activation of heat-shock proteins such as HSP-47, while avoiding irreversible denaturation or necrosis.^6^ This “controlled thermal-shock window” enables immediate recoil followed by progressive matrix reorganization over subsequent weeks. Because the dermis contains substantially more water than adipose tissue, low-temperature dielectric heating preferentially engages dermal–septal structures while minimizing thermal impact on subcutaneous fat. Classical analyses confirm that human adipose tissue is characterized by relatively low water and electrolyte content, further supporting its resistance to dielectric heating.^7^

Findings from oncologic hyperthermia research additionally show that controlled temperatures in the mid-40 °C range induce cellular stress responses and mild protein unfolding without destructive coagulation, aligning closely with the thermal behavior targeted by low-temperature dielectric techniques.^8^

Given this physiologic foundation, the DLTD system—when applied at lower-intensity settings—is hypothesized to promote effective improvement of nasolabial folds and marionette lines by facilitating selective dermal–septal tightening, collagen recoil, and FSN retention, while avoiding the high-temperature risks characteristic of HIFU and RF. The present study evaluates clinical outcomes following a single session delivered using the DLTD system, applied along the midface-to-lower-face axis in women aged 35–65 years, using validated wrinkle grading scales, 3D vector analysis, and blinded aesthetic evaluations.

## Methods

### Study population

This study was conducted in accordance with the Declaration of Helsinki, and written informed consent was obtained from all participants. A total of 32 female adults aged 35–65 years with clinically visible nasolabial folds and marionette lines were enrolled. Exclusion criteria included recent energy-based facial procedures, dermal fillers, pregnancy or lactation, active dermatitis or infection, autoimmune or connective-tissue disorders, systemic retinoid use, or a history of keloid formation.

### Pre-treatment assessment

Baseline photographs and 3D imaging were obtained under standardized illumination, camera distance, and facial stabilization. Two board-certified dermatologists independently graded baseline wrinkle severity using the Wrinkle Severity Rating Scale (WSRS) for nasolabial folds and Merz Marionette Line Scale for marionette lines. Anatomical mapping of the midface–perioral–lower-face region identified zygomatic support vectors, nasolabial fold trajectories, and marionette descent lines to guide treatment directionality.

### Treatment procedure

All participants received a single treatment session using Alltite® (Innoxus Inc., Anyang, Korea), a platform that employs the DLTD system. Treatments were applied along a continuous anatomical pathway from the midface to the lower face, including the zygomatic support zone, the nasolabial fold corridor, and the marionette region.

**Figure 2:**
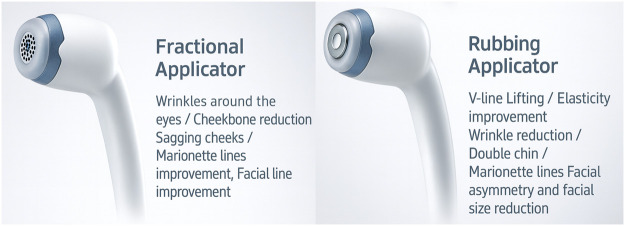
Functional design and clinical indications of the two DLTD applicators. Fractional Applicator—optimized for superficial and mid-dermal engagement, suitable for periorbital wrinkles, cheekbone contour softening, sagging cheeks, marionette line refinement, and general facial-line improvement. Rubbing Applicator—designed for broader, deeper dielectric delivery for V-line contour definition, elasticity enhancement, wrinkle reduction, double-chin improvement, marionette-line correction, and reduction of facial asymmetry or overall facial width.

The skin was cleansed with alcohol, and a uniform layer of coupling medium was applied. Two specialized applicators were used according to local tissue characteristics: fractional applicator and rubbing applicator (Figure 2).

Each session consisted of approximately 400 total shots delivered using a combination of both applicators, with allocation proportional to regional wrinkle severity and the intended support vectors.

Treatment modules were moved with consistent pressure and velocity along collagen-oriented vectors: lateral-to-medial strokes over the midface, oblique or vertical strokes following the nasolabial fold, and superior-to-inferior strokes along the marionette axis. Integrated epidermal cooling maintained stable surface temperatures. No anesthesia, post-procedure medication, or cooling was required. Participants were advised to avoid heat exposure and irritant topical products for 48 h.

### Assessment framework

All subjects were evaluated at Baseline, immediately post-treatment, Week 4, and Week 8 under identical imaging conditions, including fixed camera distance, standardized illumination, and controlled facial positioning. All enrolled participants completed every follow-up visit. Clinical ratings were performed by two independent board-certified dermatologists who were blinded to the assessment timepoints.

### Primary endpoints

Primary outcomes were wrinkle-severity scales targeting nasolabial folds and marionette lines. Nasolabial fold depth was rated using Wrinkle Severity Rating Scale (WSRS), which evaluates fold prominence and contour disruption. Marionette line severity was assessed using Merz Marionette Line Scale (Merz Scale), which grades the depth and downward projection of the perioral groove. Both dermatologists scored each timepoint independently, with discrepancies resolved by consensus.

### Secondary endpoints

Secondary outcomes consisted of global aesthetic ratings and three-dimensional structural measurements. The Investigator Global Aesthetic Improvement Scale (I-GAIS) and the Patient GAIS (P-GAIS) were administered at Week 8 to capture physician- and patient-perceived overall improvement compared to Baseline.

Objective soft-tissue displacement was quantified using a standardized 3D stereophotogrammetric vector analysis. Facial meshes were aligned via rigid registration using stable bony landmarks (infraorbital rim, alar base, zygomatic arch), and displacement vectors were calculated by comparing predefined soft-tissue coordinates over time. Midface elevation was defined as superior displacement of the malar region of interest, whereas marionette-line improvement was defined as reduced inferior vector shift along the perioral descent pathway. These geometric metrics complement wrinkle-severity scores by quantifying tissue-support restoration.

Together, these multimodal assessments enabled comprehensive evaluation of both wrinkle-specific improvements and broader structural changes following treatment.

### Safety assessment

Safety monitoring was conducted at every study visit—Immediately post-treatment, Week 4, and Week 8—to identify any treatment-related adverse events. At each evaluation, investigators examined the treated regions for erythema, edema, burns, blistering, nodules, sensory disturbances, and contour irregularities. Participants were also asked to report any interval symptoms between visits.

Procedural pain was assessed once, immediately after treatment, using a standardized 0–10 numeric rating scale, as no prophylactic anesthesia or cooling was applied. This allowed direct evaluation of true procedural discomfort.

## Results

### Primary results

A single DLTD session produced progressive and clinically meaningful improvement in wrinkle severity across both primary endpoints. WSRS decreased immediately after treatment (3.1 ± 0.4 to 2.8 ± 0.4) and continued to improve through Week 8, reaching 2.0 ± 0.4 (35.5% reduction). Merz Scale followed a similar but slightly milder trajectory, improving from 2.6 ± 0.5 at baseline to 2.4 ± 0.4 immediately post-treatment and demonstrating continued improvement over follow-up, reaching 1.9 ± 0.3 (26.9% reduction) by Week 8 (Table 1). These early changes correspond to acute collagen recoil, with later improvements reflecting ongoing dermal–septal remodeling.

**Table 1:** WSRS and Merz severity scores over time.

Timepoint	WSRS	Change	Improvement (%)	Merz	Change	Improvement (%)
Baseline	3.1 ± 0.4	–	–	2.6 ± 0.5	–	–
Immediate	2.8 ± 0.4	−0.3	9.7%	2.4 ± 0.4	−0.2	7.6%
4 Weeks	2.3 ± 0.4	−0.8	25.8%	2.0 ± 0.4	−0.6	23.1%
8 Weeks	2.0 ± 0.4	−1.1	35.5%	1.9 ± 0.3	−0.7	26.9%

**Figure 3:**
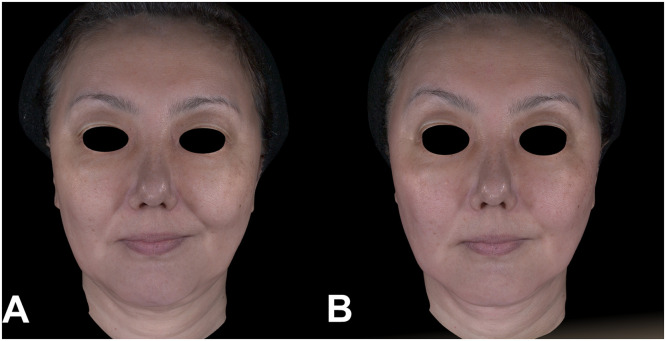
Clinical photographs of a 45-year-old female patient following DLTD treatment. (A) Baseline and (B) Week 8 after a single DLTD session (400 shots). Compared with baseline, the Week-8 image shows reduced prominence of the nasolabial folds and smoother contour of the marionette-line region.

**Figure 4:**
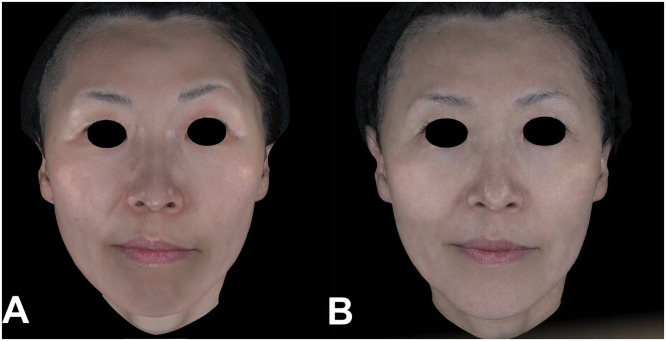
Clinical photographs of a 54-year-old female patient before and immediately after DLTD treatment. (A) Baseline and (B) Week 8 after one DLTD session (400 shots). The Week-8 photograph demonstrates a visible decrease in nasolabial-fold depth and improved definition of the marionette-line contour relative to baseline.

Representative clinical cases demonstrated the same pattern of improvement, with visible reduction in nasolabial-fold depth and marionette-line prominence at Week 8 in both a younger patient (Figure 3 and an older patient (Figure 4).

Both wrinkle regions showed consistent improvement across timepoints, with nasolabial folds demonstrating slightly greater responsiveness than marionette lines. Improvements at 4 and 8 weeks reflect sustained remodeling rather than transient post-procedural changes.

### Secondary results

Three-dimensional vector analysis confirmed structural elevation and perioral descent correction. Midface vertical elevation increased from +0.6 ± 0.2 mm immediately post-treatment to +1.9 ± 0.5 mm at Week 8. Inferior marionette deflection decreased from −0.4 ± 0.1 mm immediately to −1.2 ± 0.3 mm at Week 8, demonstrating reinforcement of midface and lower-face support (Table 2).

**Table 2:** Three-dimensional vector outcomes: midface elevation and marionette inferior-vector reduction.

Timepoint	Midface vertical elevation (mm)	Marionette descent reduction (mm)
Immediate	+0.6 ± 0.2	−0.4 ± 0.1
4 Weeks	+1.4 ± 0.4	−1.0 ± 0.3
8 Weeks	+1.9 ± 0.5	−1.2 ± 0.3

Global aesthetic improvement was high. At Week 8, 95% of investigator ratings and 92% of patient ratings fell within the “Improved” or better categories on the I-GAIS and P-GAIS, respectively (Table 3). These findings align closely with the objectively measured vector-based elevation and wrinkle-severity reductions.

**Table 3:** Aesthetic improvement (GAIS – Week 8).

GAIS category	Investigator (%)	Patient (%)
Very much improved	22%	18%
Much improved	45%	40%
Improved	28%	34%
No change	5%	7%
Worse	0%	1%

### Safety

No serious adverse events occurred. No participant exhibited burns, blistering, edema, prolonged erythema, sensory disturbances, nodules, or contour irregularities (Table 4). All events were mild and transient.

**Table 4:** Adverse events summary.

Event type	Incidence	Severity	Duration
Erythema	3/30 (10%)	Mild	<30 min
Warmth/Tingling	6/30 (20%)	Mild	Immediate only
Edema, burns, blistering, sensory change	0/30	–	–
Contour irregularities/Fat atrophy	0/30	–	–

Pain levels were exceptionally low. Immediately after treatment, the mean pain score was 0.4 ± 0.6 on a 0–10 numeric rating scale. Most participants described the sensation as “warm” or “slightly tingling,” and no subject required topical anesthesia or post-treatment analgesics.

Overall, the DLTD procedure was well tolerated, required no anesthesia, and produced no downtime.

### Other findings

**Figure 5:**
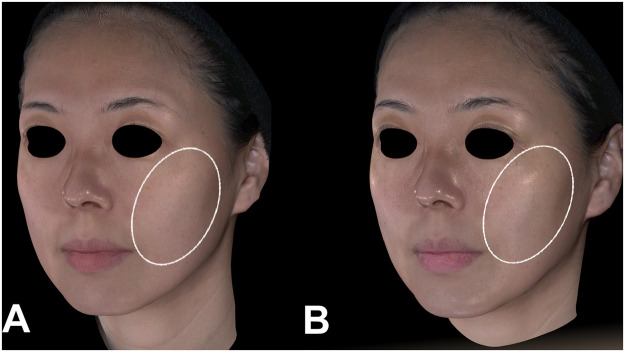
Clinical photographs of a 33-year-old female patient before and immediately after DLTD treatment. (A) Baseline and (B) immediately after a single treatment session (400 shots). The immediate post-treatment image shows visible improvement in skin laxity and elasticity, along with increased surface smoothness in the mid-to-lower facial region, particularly within the circled area.

In addition to wrinkle-specific outcomes, many participants reported subjective improvement in skin laxity, particularly along the mid-to-lower-face corridor. Immediately after treatment, 83% of subjects noted a sensation of firmer skin or improved facial support. By Week 8, 89% reported noticeable enhancement in overall skin tightness. Figure 5 shows enhanced mid-to-lower facial tightness immediately after a single DLTD session. These findings are consistent with the immediate thermal-recoil response and the progressive retension of the fibro-septal network observed in the 3D vector analysis.

## Discussion

The present study demonstrates that a single session of dermis layer targeted dielectric heating (DLTD) produces consistent improvement in nasolabial folds and marionette lines, accompanied by measurable restoration of mid-to-lower facial support. Improvements in WSRS and Merz scores, together with progressive three-dimensional vector elevation, indicate that DLTD influences not only superficial dermal texture but also deeper load-bearing connective tissue structures. The magnitude and directionality of these changes—particularly the +1.9 mm midface elevation and −1.2 mm reduction in inferior marionette displacement at Week 8—are consistent with biomechanical retension of the fibro-septal network (FSN) and retinacula cutis (RC), which are recognized determinants of age-related facial descent.^3^

### DLTD and other thermal modalities

**Figure 6:**
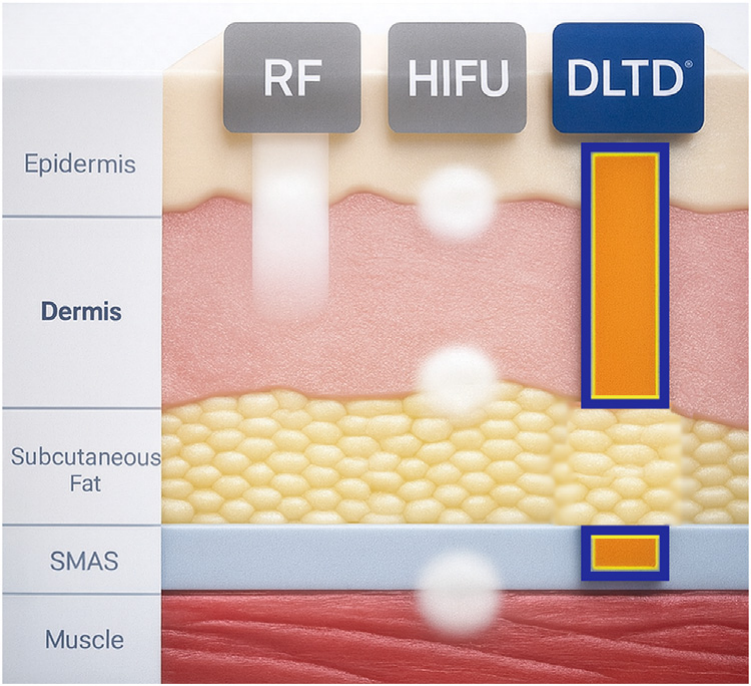
Comparative depth of RF, HIFU, and DLTD. RF concentrates peak temperature superficially within the epidermis/upper dermis, HIFU produces discrete high-temperature coagulation zones at the SMAS level, whereas DLTD delivers selective volumetric heating spanning the mid-dermis, fibro-septal network, and superficial SMAS.

Interpretation of these findings warrants comparison of DLTD with established thermal rejuvenation modalities, particularly radiofrequency (RF) and high-intensity focused ultrasound (HIFU), given their differing mechanisms of energy delivery and tissue interaction (Figure 6).

Conventional RF systems rely on impedance-dependent Joule heating, generating temperature gradients that peak at the epidermal–dermal junction. This superficial concentration limits uniform energy delivery to structurally critical mid-dermal and septal layers and accounts for the predominance of texture improvement and mild laxity reduction rather than consistent vertical elevation.^9^, ^10^, ^11^, ^12^ Histologic evaluations further demonstrate collagen reorganization and dermal remodeling following non-ablative RF treatment, indicating that RF-induced rejuvenation reflects a spectrum of tissue remodeling rather than isolated neocollagenesis.^11^^,^^13^

At the opposite end of the thermal spectrum, HIFU delivers acoustically focused energy that produces discrete microcoagulation zones, typically at the level of the superficial musculoaponeurotic system (SMAS), with peak temperatures exceeding the coagulative threshold.^5^ While this mechanism enables focal tissue contraction, the discontinuous nature of energy delivery is associated with procedural pain, edema, and susceptibility of adjacent subcutaneous fat, particularly in regions with variable adipose thickness.^14^, ^15^, ^16^

DLTD occupies a mechanistically distinct intermediate position. Through dielectric dipole oscillation, DLTD achieves volumetric energy deposition governed by tissue permittivity and water content, resulting in preferential heating of the mid-dermis, fibro-septal network (FSN), and superficial SMAS within a sub-coagulative thermal range. This selective absorption pattern is supported by dielectric modeling and Ultra high frequency physics literature,^1^^,^^4^^,^^8^ as well as classical analyses demonstrating the low water content and relative dielectric resistance of adipose tissue ^6^. Within this controlled thermal window, collagen triple-helix recoil and early fiber tightening may occur without irreversible denaturation or tissue necrosis.^2^

Accordingly, the immediate wrinkle softening observed in this study is consistent with rapid collagen recoil, whereas continued improvement through Week 8 reflects delayed extracellular matrix reorganization and progressive FSN retension. The resulting vector-based lifting pattern aligns more closely with connective-tissue retension than with adipose contraction or thermal adipolysis, consistent with RC–FSN aging models in which deterioration of these anchoring structures drives inferior malar migration and perioral descent.^3^

### Thermal and injectable biostimulatory treatments

Device-based dielectric heating and injectable biostimulatory treatments aim to improve skin laxity through collagen remodeling, yet they do so via fundamentally different biophysical triggers and tissue targets. DLTD induces remodeling through temperature-dependent collagen recoil and downstream fibroblast activation generated by volumetric dielectric energy deposition. Because energy absorption is governed by tissue permittivity and hydration, the primary targets of DLTD are water-rich connective tissue structures, including the mid-dermis, FSN, and superficial SMAS. Accordingly, the depth of action is defined by intrinsic tissue properties rather than by the placement of an exogenous material.^1^^,^^2^^,^^4^^,^^8^

In contrast, injectable biostimulatory agents initiate remodeling through material–tissue interactions within a predefined injection plane. Hyperdiluted calcium hydroxylapatite (CaHA) functions as a biostimulatory scaffold that activates fibroblasts via particle-associated mechanotransduction and has been widely adopted for skin quality improvement and tightening, with reported effects spanning the deep dermis to superficial subdermis depending on dilution and injection depth.^17^, ^18^, ^19^ Poly-L-lactic acid (PLLA) induces collagen deposition through a foreign-body inflammatory cascade, with tissue response determined by particle persistence and host inflammatory reactivity.^20^ Thus, for injectables, both the target layer and depth of action are operator-defined and dependent on injection technique.

These mechanistic differences result in distinct tissue remodeling patterns. DLTD delivers a field-driven thermal stimulus and therefore promotes remodeling distributed along existing dermal and fibro-septal architecture rather than localized to discrete foci. Injectable biostimulators, by contrast, can produce spatially heterogeneous remodeling that mirrors the distribution of injected material; while advantageous for volumization or region-specific tightening, such heterogeneity may manifest as focal nodularity or localized fibrosis, particularly in the context of PLLA.^20^^,^^21^ From a long-term perspective, the critical distinction is whether remodeling alters tissue planes diffusely or introduces focal disruptions that may influence future surgical access.

Against this background, while neocollagenesis remains a principal mechanism underlying DLTD, it is essential to recognize that all collagen-remodeling interventions—thermal or injectable—exist on a continuum of tissue responses that includes some degree of dermal and/or subdermal fibrosis.

### Fibrosis and tissue remodeling

Histologic studies of non-ablative thermal stimulation consistently demonstrate collagen reorganization, increased matrix density, and fibroblast activation following controlled heating, indicating that regenerative remodeling and fibrosis represent biologically continuous processes rather than mutually exclusive outcomes.^11^^,^^13^ The clinical relevance of fibrosis therefore depends not on its presence alone, but on its distribution, depth, and relationship to native tissue architecture.

In DLTD, collagen remodeling occurs without particulate deposition or focal coagulative injury and is driven by temperature-dependent molecular reorganization within native connective tissue frameworks. As a result, remodeling is expected to follow existing dermal and fibro-septal planes rather than forming discrete fibrotic nodules. In contrast, injectable biostimulatory agents may induce fibrosis that is spatially heterogeneous and influenced by particle distribution and injection plane, with documented cases of nodular fibrosis and foreign-body reactions, particularly following PLLA treatment.^20^^,^^21^

### Potential impact on future facial surgery

The potential impact of prior nonsurgical aesthetic procedures on subsequent facial surgery warrants explicit consideration. Surgical literature has documented that both energy-based device treatments and injectable biostimulatory procedures can alter superficial tissue planes, increase fibrosis, and complicate subsequent facelift or neck-lift dissection.^22^^,^^23^ Intraoperative findings following nonsurgical treatments have included fibrosis, loss of normal tissue planes, and fat atrophy, all of which may increase dissection resistance and obscure anatomic landmarks.^22^

Such alterations are particularly relevant in procedures requiring precise dissection along the superficial SMAS-adjacent plane, where fibrosis may increase technical difficulty and potentially elevate the risk of injury to peripheral branches of the facial nerve.^24^ Reports examining facelift surgery after PLLA treatment illustrate this variability, with some series noting minimal interference and others describing fibrotic changes necessitating modified technique and heightened caution.^21^ Broader analyses of secondary and revision facelifts further emphasize that scarred or altered tissue planes—regardless of etiology—are a consistent source of increased surgical complexity.^25^

Within this context, the non-coagulative and non-particulate remodeling profile of DLTD —when utilized within the low-to-moderate intensity range—may theoretically reduce the likelihood of severe focal plane distortion compared with high-temperature thermal coagulation or particle-associated fibrosis. Nevertheless, even with these controlled energy parameters, altered tissue planes cannot be excluded. Accordingly, patients who may desire surgical facial rejuvenation in the future—particularly younger individuals or those pursuing staged aesthetic interventions—should be carefully selected, and informed consent should explicitly address the potential long-term impact of these specific nonsurgical treatments on subsequent surgery.

### Limitations and future directions

Several limitations should be acknowledged. The absence of a control group limits direct comparison with RF, HIFU, or injectable biostimulatory treatments, and the 8-week follow-up reflects only early remodeling dynamics. Although three-dimensional vector analysis quantified positional changes, it did not directly assess collagen orientation, FSN stiffness, or tissue elasticity.

Future studies should incorporate randomized split-face designs, extended follow-up periods of 6–12 months or longer, and advanced imaging modalities such as elastography or shear-wave stiffness mapping to further elucidate the biomechanical and architectural effects of DLTD on the dermal–septal framework. Comparative investigations evaluating DLTD in combination with injectable biostimulatory agents may also clarify the role of sequential or synergistic treatment strategies.

## Conclusion

This study demonstrates quantifiable clinical and structural changes following a single session of dermis layer targeted dielectric heating (DLTD) delivered at low-to-moderate intensity settings for mid- and lower-face rejuvenation, interpreted in relation to existing thermal and injectable rejuvenation modalities. Using validated wrinkle severity scales and three-dimensional vector analysis, reductions in nasolabial fold and marionette line severity were observed in parallel with measurable vertical midface elevation and reduced inferior marionette displacement, indicating functional improvement of mid–lower facial support.

The present analysis suggests that the observed clinical improvements extend beyond superficial wrinkle attenuation and are consistent with structural remodeling involving dermal and fibro-septal components. By examining the depth of action and mode of energy delivery of DLTD —specifically its efficacy within moderate energy parameters—in comparison with high-temperature thermal devices and injectable biostimulatory treatments, this study contextualized DLTD within a spectrum of collagen-remodeling interventions characterized by differing mechanisms, tissue targets, and remodeling patterns.

In addition, this study addressed tissue-level responses that may accompany collagen remodeling therapies, including the spectrum of dermal and subdermal fibrotic changes reported across thermal and biostimulatory modalities, and reviewed their potential implications for subsequent facial surgery based on existing surgical literature. This integrative approach highlights the importance of evaluating non-surgical facial rejuvenation techniques not only in terms of short-term aesthetic outcomes, but also with regard to underlying tissue remodeling and longer-term structural considerations.

Taken together, the findings of this study demonstrate that DLTD is associated with measurable clinical and structural improvements in mid- and lower-face aging and provide a framework for interpreting its effects relative to other energy-based and injectable rejuvenation strategies within the context of connective tissue remodeling.

## Ethical statement

This study was conducted in accordance with the ethical principles of the Declaration of Helsinki. Informed consent was obtained from all participants. No identifying patient information was collected or used.

## Informed consent

Informed consent was obtained from all participants, with full disclosure of the study’s purpose, risks, and confidentiality.

## Photo consent

Written informed consent for publication of clinical photographs was obtained from all participants.

## Author contributions

All authors have reviewed and approved the article for submission. Conceptualization: Kyuho Yi, Ohdeog Kwon. Writing—Original Draft: Yerin Park, Ohdeog Kwon. Writing—Review & Editing: Ohara Natsue, Kaveh Karandish, Eric Sooyoung Ahn, Ti Jo Tsay, Kyuho Yi. Visualization: Yerin Park. Supervision: Kyuho Yi.

## Declaration of competing interest

The authors declare that they have no conflicts of interest to disclose.
